# A novel D2D–MEC method for enhanced computation capability in cellular networks

**DOI:** 10.1038/s41598-021-96284-w

**Published:** 2021-08-19

**Authors:** Xiangyan Liu, Jianhong Zheng, Meng Zhang, Yang Li, Rui Wang, Yun He

**Affiliations:** 1 School of Communication and Information Engineering, Chongqing University of Posts and Telecommunications, Chongqing, 400065 China; 2 Cyberspace Security Research Institute of China Electronics Technology Group, Chengdu, 610041 China; 3 Department of Electronic communication Engineering, Yuxi Normal University, Yunnan, 653100 China

**Keywords:** Electrical and electronic engineering, Information technology

## Abstract

Device-to-device (D2D) communications and mobile edge computing (MEC) used to resolve traffic overload problems is a trend in the cellular network. By jointly considering the computation capability and the maximum delay, resource-constrained terminals offload parts of their computation-intensive tasks to one nearby device via a D2D connection or an edge server deployed at a base station via a cellular connection. In this paper, a novel method of cellular D2D–MEC system is proposed, which enables task offloading and resource allocation meanwhile improving the execution efficiency of each device with a low latency. We consider the partial offloading strategy and divide the task into local and remote computing, both of which can be executed in parallel through different computational modes. Instead of allocating system resources from a macroscopic view, we innovatively study both the task offloading strategy and the computing efficiency of each device from a microscopic perspective. By taking both task offloading policy and computation resource allocation into consideration, the optimization problem is formulated as that of maximized computing efficiency. As the formulated problem is a mixed-integer non-linear problem, we thus propose a two-phase heuristic algorithm by jointly considering helper selection and computation resources allocation. In the first phase, we obtain the suboptimal helper selection policy. In the second phase, the MEC computation resources allocation strategy is achieved. The proposed low complexity dichotomy algorithm (LCDA) is used to match the subtask-helper pair. The simulation results demonstrate the superiority of the proposed D2D-enhanced MEC system over some traditional D2D–MEC algorithms.

## Introduction

In recent years, innovative applications such as artificial intelligence, face recognition and interactive games promote the access of large-scale devices^1^. The fundamentally new 5G key physical layer technologies such as multi-input multi-output (MIMO), non-orthogonal multiple-access (NOMA) and full-duplex (FD) transmission for radio access networks further increase the network capacity as well as the number of accessible devices^2^. According to the latest forecast report provided by Cisco, by 2023, the number of terminals connected to the world will reach nearly 30 billion^3^. The surge of emerging applications accelerates the improvement of computation and network capacity compared to traditional wireless networks. The European Telecommunications Standard Institute (ETSI) took the lead in putting forward the concept of Mobile Edge Computing (MEC) in 2014, which then published the white paper of MEC technology^4^. Through MEC, the shortcomings of traditional mobile cloud computing can be effectively overcome, cloud computing capabilities can be expanded from centralized clouds to the edge of a networks, and the processing capabilities can be enhanced, providing rich and low-latency computing for ultra-dense networks (UDN), and meanwhile avoiding the problem of long latency and overload of the core networks^5,6^.

However, compared with cloud servers, edge servers have relatively limited computing resources^7^. In future scenarios of Massive Machine Type Communications(MMTC) or Internet of Things (IoT), when tasks are executed through MEC servers with limited computing resources for a large number of mobile users at the same time, the servers may become overloaded, which will reduce the performance of the wireless system. Collaborative computing among users is an effective way to extend the execution capability of MEC. Device-to-Device (D2D) communication is widely concerned because it can share channel resources with cellular networks and achieve a higher spectrum utilization as well as resource scheduling^8^. On the other hand, the computation-intensive tasks have higher requirements for highly reliable accesses and low-delay processing, and computation resources can be further expanded through joint communication between D2D communication and MEC^9^.

Offloading strategy speeds up the computing process and prolongs the battery life of terminal equipment^10^. How to efficiently realize task offloading and resource allocation is a hot issue in the current research on D2D-enhanced MEC system. Devices needs to make offloading decisions according to the tasks requirements and decide whether the tasks should be executed locally or offloaded to a third-party device nearby via D2D connection or an edge server deployed in a base station via cellular connection^11–13^. The information-centric IoT is considered in the works in^14^, in which the partial offloading model is considered and the task is divided into multiple subtasks. The computing resources of the BS are used to manage the control information to find subtask-helper pairs for the devices with several precedence-constrained subtasks^14^. Unlike^14^, a system model is proposed in Research^15^ for cooperative mobile edge computing, in which a device social graph model is developed to capture the social relationship among devices. The task dependency graph is closely related to the social graph, which facilitates flexible choices of task execution approaches^15^. Game-theoretic models for device-enhanced MEC offloading with a large number of UEs have been investigated in Study^16^. More specifically, the problem of offloading decision-making among users has been formulated as a sequential game, which achieves the Nash equilibrium^16^.

Both device access and delay requirement should be considered in offloading decisions because they are important factors affecting the quality of experience (QoE) of users^17,18^. The variability of mobile device capabilities and user preferences is leveraged in Reference^19^, formulating the system utility metric as a measure of QoE based on task completion time and energy consumption of a mobile device^19^. In^20^, a task model is considered to minimize the latency of local users, who have multiple independent computation tasks that can be executed in parallel but can not be further partitioned. The tasks can be offloaded to helpers and the results can be downloaded from them over prescheduled time slots enabled by the proposed TDMA-based communications^20^. A system is put forward in the works in^21^ from the perspective of holism. Four levels of heterogeneous cloud units with various hardware capabilities are employed in the system, including the D2D communication units and the edge cloud units^21^, through which a higher efficiency is achieved in terms of offloading, congestion, coverage and latency.

As the delay and energy consumption are affected by the offloading schemes of mobile users, the D2D–MEC systems have been investigated in recent works from the perspective of total computation latency minimization^20,22^, total energy consumption minimization^23–33^ and the tradeoff between the two objectives^16,34–38^. A time division multiple access (TDMA) transmission protocol is proposed in^22^ for minimizing the total computation latency of mobile devices with multi-helpers^22^. Like^22^, it is supposed in Reference^23^ that each device can be offloaded to multi-helpers, jointly considering helper selection, communication and computation resources allocation^23^. Kai et al. used Initial Task Allocation (ITA) algorithm to maximize the number of executed tasks, and the problem of global energy minimization was tackled while maintaining the maximum number of executed tasks^25^. To enable the minimization of energy consumption, an adaptive offloading scheme is proposed in the work of^28^, and the helper controls the offloading process based on a predicted helpers’ CPU-idling profile that specifies the amount of computation resource available for co-computing^28^. An optimization problem is formulated in^30^ to minimize the time-average energy consumption of task execution of all mobile devices, taking into consideration the incentive constraints resulted from the prevention of over-exploiting and free-riding behaviors. A Lyapunov optimization method based on online task offloading is derived accordingly^30^. The Lyapunov optimization technology framework is also leveraged in Reference^31^ to solve the problem of stochastic optimization, which is formulated as a problem of continuous arrival task offloading of mobile devices^31^. A two-step algorithm has been proposed in Study^32^, and delay-sensitive tasks are processed in the first step, while tasks of users with energy restrictions are processed in the second step. The proper offloading destination is found by the MEC server through the maximum matching with the minimum cost graph algorithm^32^. Except for traditional methods, a Q-learning algorithm and a deep Q-network algorithm are applied in^27^, meanwhile the long-term energy consumption in continuous time is minimized via reinforcement learning in^33^.

To maximize a weighted sum of reductions in task completion time and energy consumption, a MEC-enabled multi-cell wireless network is considered in^34^ to assist mobile users in executing computation-intensive tasks^34^. A latency and energy consumption minimization scenario is proposed in Work^37^ for a system with one BS. The formulated problem is transformed into a sub-problem of computation offloading and that of resource allocation, which are respectively solved through the Kuhn-Munkres algorithm and the Lagrangian dual method^37^. A dynamic social-motivated computation offloading method has been proposed in Study^38^, through which the task computation latency and energy consumption are jointly minimized. A Lyapunov optimization-based method and a drift-plus-penalty algorithm are used to solve this problem^38^.

A framework consisting of MEC and cache-enabled D2D communication is enabled in^39^ to enhance computing offloading and caching capabilities^39^. Like in^39^, the task caching in the context of a device-enhanced MEC system has been examined in Study^40^. Sun W et al.^41^ propose a Stackelberg game-based incentive mechanism to encourage other devices to help a device finish the computing tasks, then through a graph-based algorithm, the optimal task assignment solution is found to improve the task processing efficiency^41^. A basic three-node MEC system with two UEs is considered in the works in^42^, whereby one UE needs computation resources and the other is the helper/relay^42^. Moreover, one AP node is attached to one MEC server. A four-slot protocol is proposed to enable energy-efficient device-enhanced MEC. Besides, federated learning is involved in D2D-assisted MEC networks to lower the communication cost^43^. The benefits of internet service providers(ISPs) are maximized in Reference^44^. Finally, we provide a comparison of the pros and cons in the recent research in Table 1.

**Table 1:** The comparison of the related studies.

Ref.	Purpose	Year	Advantages	Shortcomings
^5^	Minimizing the response delay of requests.	2020	A multi-server system is taken into consideration	Regardless of the allocation of bandwidth
^10^	Maximizing the supported links.	2020	Considering resource allocation and power control together	HetNet is not considered
^14^	Minimizing the time and financial cost for the users	2019	Taking into consideration the precedence-constrained subtasks	The BS does not participate in the computing process
^15^	Reduce the computation cost.	2018	Facilitate flexible choices of task execution approaches	Extra resources are needed to maintain socially motivated cooperation
^16^	Decrease the task execution delay and the energy consumption.	2018	The offloading scheme can meet a Nash equilibrium of the formulated game	Only one mobile user updates its offloading decision in each decision slot
^19^	Maximizing the system utility by the improvement in QoE.	2017	Improve the QoE of each mobile device	Without considering the maximize time latency
^20^	Minimizing the computation delay of local users.	2018	A device can offload its tasks to several nearby end devices	Only one user is considered.
^21^	Achieve higher efficiency	2018	A new four levels of heterogeneous cloud is proposed.	The new heterogeneous cloud is hard to deploy
^23^	Minimizing the overall energy consumption	2020	The tasks of each device can be offloaded to multi-helper	The network control factor is not explicitly formulated
^25^	Maximizing the number of executed tasks.	2019	The number of access devices has improved	Only one BS is taken into consideration
^27^	Minimizing the energy cost	2020	MEC with cache-enabled D2D communications is proposed	The complexity of the algorithm is high
^28^	Minimizing the energy consumption.	2018	Applying computation prediction at edge devices	The adaptive offloading of computation tasks is not practical
^29^	Minimizing the energy consumption of equipments.	2017	Deals with the D2D crowd task assignment problem	The energy-efficiency of the D2D clusters is not considered.
^30^	Minimizing the energy consumption of mobile devices	2016	Computation resources of mobile devices can be shared through devices	The network control factor is not explicitly formulated in the scheme
^31^	Minimizing the energy consumption for mobile devices	2019	Handle the continuous arrival tasks of mobile devices	Communication overhead is neglected
^32^	Minimizing the energy consumption of the task execution	2020	The scheme is designed for devices with delay-sensitive tasks or low energy	The fairness among devices is not considered
^33^	Minimizing energy consumption	2020	The long-term energy consumption in continuous time is minimized	Only idle users can be helpers
^34^	Maximizing reductions in task completion time and energy consumption	2019	Users can improve the efficiency of task execution	The performance discrepancy between multi-server is not considered
^37^	Minimizing execution latency and energy consumption	2018	The optimal tradeoff between execution latency and energy consumption	The task execution relationship between devices is not considered
^38^	Minimizing the task computation latency and the energy consumption	2019	Social-motivated computation is used in the D2D–MEC system	Did not specifically evaluate security metrics
^40^	Maximizing a utility defined based on delay and energy consumption	2019	Integrate caching into D2D-aided computing Networks	Caching is conducted in an offline manner
^41^	Maximizing the computing profits of task publishers	2019	Utilizing the computing resources of idle devices to enhance computing capability	Ignore the profits of the helper device
^42^	Improving energy efficiency in mobile computing	2018	A novel four-slot protocol is propose	The simple evaluation topology included only two user equipment
^43^	Lowering the communication cost	2021	Federated Learning is involved in D2D-assisted MEC networks	It’s hard to guarantee the robustness of the system
^44^	Maximizing the network management profit	2021	Maximize the benefits of ISPs.	The fog node is not specified

The problems formulated from the macro perspective are mainly solved in the aforementioned studies, i.e., the total task completion time, energy consumption or system capacity. However, the computing efficiency of independent devices in the D2D–MEC system has not been considered. Besides, a binary offloading strategy is considered in most studies to execute tasks in the system. In the studies of partial offloading, either the high-performance helpers with a few tasks are ignored or the computing resources of devices and the edge server are neglected. Inspired by this, we integrate D2D communication with the MEC system and propose a novel D2D–MEC method. To facilitate our analysis, we make the following reasonable assumptions throughout this paper.

1. All devices in the network are linked to and can communicate with the edge server. Besides, devices can communicate with each other. Once the devices complete their tasks within the time limit, the remaining computing resources can be used to continue helping other devices, which is similar to^14^. Only if the devices fail to complete the tasks on time will they offload the tasks to the helper or the edge server.
2. Each device has a computationally intensive and time-sensitive task that needs to be completed within a certain time, which can be segmented. As the works in^41^, the task can be executed locally, offloaded to one of the helper devices nearby or executed by offloading to the edge server. When a task arrives, each device will first predict whether the local computing resources are sufficient to complete it within a specified delay based on its computational capability. If so, the task can be executed locally. Otherwise, according to the proposed algorithm, the incomplete part will be offloaded to the helper device or the edge server.
3. As the works in^39^, in the task execution process, the maximum time required by all devices is constant and equal, i.e., synchronous execution. Only when a task is completed and can the next be continued in execution simultaneously among devices. Asynchronous task processing deserves further investigation.

We adopt a partial offloading strategy and make full use of the computing resources within a specified delay, so as to improve the number of supported devices and their computing efficiency in the system. Since users tend to execute tasks through their own devices, a clustering algorithm is adopted to divide a task into two subtasks considering the users’ computing capability. One subtask is executed locally, and the other is offloaded to a helper device or an edge server. LCDA is proposed to match the subtask-helper pair. Then, the system limitations are analyzed in terms of the size of the task, the tolerance time and the number of access devices. Besides, task execution, task offloading and computation resource allocation can be completed within the time constraints. Computing efficiency can be increased through the D2D-assisted MEC system effectively, and the computation resources in both the edge server and the devices are highly utilized in the system. The main contributions to this work are summarized as follows.

1. To improve the network capability and computing capability, we propose a collaborative D2D-assisted MEC system that supports multi-level task offloading and resource allocation. In particular, we divide the task into two subtasks through partial offloading, which can be separately executed locally and remotely, followed by the offloading decision of each device from the microscopic point of view instead of the previous task assignment from the macroscopic view. Finally, the computation resource allocation of the MEC server is given. The problem is proposed as a mixed-integer non-linear problem, which is resolved through a two-phase heuristic algorithm.
2. To increase the number of access devices and achieve the maximum task execution capability, the LCDA algorithm is proposed to match the subtask-helper pair in the system. The MEC server guarantees that the remaining subtasks can be completed. The simulation results show that in different profiles (such as the number of devices, time constraints and the size of the intensive tasks), the algorithm has a superior performance with a lower time and space complexity, which effectively improves the computing capacity of the D2D–MEC system with time constraints.
3. To verify the practicability of the proposed model and the heuristic algorithm, we use improved greedy algorithm^41^, initial task assignment algorithm^25^, bipartite graph matching algorithm^14^ and efficient delay-aware offloading scheme^32^ as the benchmark comparisons of the proposed algorithm. Compared with the four traditional D2D–MEC algorithms, the results corroborate the superior performance of the proposed scheme in the scenario with strict time constraints.

The remainders of this paper are organized as follows. The system model, problem formulation and problem solution are introduced in "Method" section. Simulation results are given in "Simulation results" section, and conclusions are given in "Conclusions" section.

## Method

In this section, we introduce the D2D-enhanced MEC system, followed by the communication and computation model. The computation model is structured in the following three modes: Local execution mode, Local+D2D execution mode and Local+D2D+Edge execution mode. Then, depending on these three modes, we propose the optimization problem to find the solution to maximize the computational efficiency of the devices. Finally, three algorithms are utilized to solve this problem.

### D2D–MEC system model

**Figure 1:**
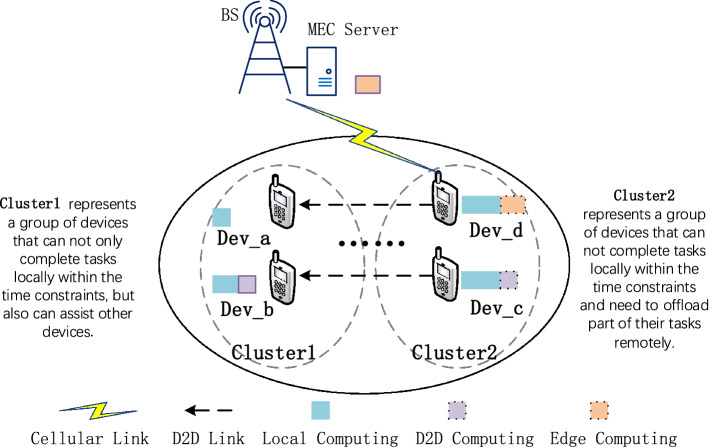
D2D-assisted MEC system. Local/D2D/Edge computing means the tasks executed via the corresponding mode.

As shown in Fig. 1, the D2D–MEC system consists of a base station(BS) equipped with an edge server and N mobile devices owned by some users. The devices are denoted by the set ${{\mathscr {N}}} \buildrel \Delta \over = \left\{ {1,2,...,N} \right\}$, which contains Cluster 1 and Cluster 2. The devices represented by Cluster1 can complete tasks locally within the time constraints and provide help to other devices. Cluster2 represents a group of devices that can not complete tasks locally within the time delay and need to offload part of their tasks remotely. They require extra computing resources from other devices via a D2D connection or the MEC server via a cellular connection. Among them, the set of devices in Cluster1 and Cluster2 are defined as ${{{\mathscr {N}}}_1} \buildrel \Delta \over = \left\{ {1,2,...,{N_1}} \right\} , {{{\mathscr {N}}}_2} \cup {{{\mathscr {N}}}_3} \buildrel \Delta \over = \left\{ {1,2,...,{N_2}{\mathrm{+ }}{N_3}} \right\}$. In Cluster1, Dev_a represents devices that cannot provide services to nearby devices via D2D connection, and Dev_b represents devices that assist adjacent devices in Cluster2, i.e., Dev_b is a helper. In Cluster2, Dev_c refers to devices in ${{\mathscr {N}}}_2$ which need additional computing resources for task execution through D2D connection, and Dev_d refers to devices in ${{\mathscr {N}}}_3$ that fail to match neighboring helpers but need to offload their tasks to the edge server. In this process, like the work in^39^, BS can get task size sequence information that the device (Cluster1) can provide through feedback and send the sequence information to the device(Cluster2) that needs help. Besides, BS can acquire the channel state information (CSI) of all devices via feedback, and the orthogonal frequency-division multiple access (OFDMA) method for channel access is adopted.

### Communication model

Each device in ${{\mathscr {N}}}_2 \cup {{\mathscr {N}}}_3$ is allocated one sub-channel for the cellular link or D2D link. Then we can denote *B* and $N_0$ as the bandwidth and the power of additive white Gaussian noise for each sub-channel respectively. If Device n chooses to offload its task remotely, using Shannon’s formula, the maximum achievable transmission rate of the D2D connection or cellular link can be expressed as

1 $$\begin{aligned} r = B{\log _2}\left( {1 + \frac{{P_n^{com}h}}{{{{\mathrm{N}}_0}}}} \right) \end{aligned}$$

where ${P}_n^{com}$ is the transmission power of one device to its nearby helper or the edge server, *h* is the channel gain, which is assumed to be known in each device and remains constant but may change from the boundary of each offloading period^45,46^. In addition, this paper considers that there are no objects and large buildings blocking radio waves in the first Fresnel region, so the LOS channel model is adopted. The notations used in this paper are summarized in Table 2.

### Computation model

For the computation model, we consider that each device n(n $\in {{\mathscr {N}}}$) has a computation task characterized by ${I}_n {\mathrm{= \{ }}{{\mathrm{D}}_{\mathrm{n}}}{\mathrm{, Ap}}{{\mathrm{p}}_{\mathrm{n}}}{\mathrm{,}} t_n^{\max } {\mathrm{\} }}$. Here ${{\mathrm{D}}_{\mathrm{n}}}$ (in bits) is the data size of the task, ${\mathrm{Ap}}{{\mathrm{p}}_{\mathrm{n}}}$ is the processing density (in CPU cycles/bit), which depends on the computational complexity of the application, ${{\mathrm{C}}_{\mathrm{n}}}$ is the total number of CPU cycles required for computing ${{\mathrm{D}}_{\mathrm{n}}}$ which can be characterized by ${{\mathrm{C}}_{\mathrm{n}}}{\mathrm{= }}{{\mathrm{D}}_{\mathrm{n}}}{\mathrm{Ap}}{{\mathrm{p}}_n}$ and $t_n^{\max }$ is the maximum tolerable latency(in second). Device n can obtain the information about ${\mathrm{D}}_{\mathrm{n}}$ and ${\mathrm{Ap}}{{\mathrm{p}}_{\mathrm{n}}}$, and they remain the same value within the time range $t_n^{\max }$.

**Table 2:** Notation.

Notation	Definition
${\mathscr {N}}$, *N*	The set/number of total devices
${{{\mathscr {N}}}_1}$, *N*1	The set/number of local computing devices
${{\mathscr {N}}}_2$, *N*2	The set/number of D2D computing devices
${{\mathscr {N}}}_3$, *N*3	The set/number of edge computing devices
${{\mathscr {A}}}_{\mathrm{n}}$	Computation offloading decision of device n
*B*	The bandwidth of one sub-channel
$$ N_0$$	Power of noise of one sub-channel
${P}_n^{com}$	The transmission power of D2D link/cellular link
${P}_n^{max}$	The maximum transmission power of D2D link/cellular link
*h*	Channel gain between D2D link or cellular link
${P}_n^{loc}$, ${P}_n^{idl}$	Power of device n in local processing/idle
${I}_n$	The computation task of device n
${\mathrm{D}}_{\mathrm{n}}$	The size of input data of ${I}_n$
${\mathrm{Ap}}{{\mathrm{p}}_{\mathrm{n}}}$	The required CPU cycles per bit of ${I}_n$
$T_n^{\max }$	Maximum time delay
${f}_n^{loc}$	Computation resources of local device
${f}_n^{dev}$	Computation resources of helper device
${f}_n^{edg}$	Computation resources assigned to device n at edge server
${\mathrm{F}}^{{\mathrm{edg}}}$	Total computational capability of the edge server
$$ {CE}_n^{loc}$$	Computing efficiency of local execution device
$$ {CE}_n^{total}$$	The CE of local computing devices of local execution and helper execution

Each device can execute its computation task locally or offload part of the task to one nearby helper device or the edge server. We denote ${{{\mathscr {A}}}_{\mathrm{n}}}{\mathrm{= }}\left\{ { {\mathrm{x}}_n , y_n ,{z}_n } \right\}$ as the offloading decision of device n. Specifically, we have ${{{\mathscr {A}}}_{\mathrm{n}}}{\mathrm{= }}\left\{ {1,0,0} \right\}$ if device n can complete its task locally. We have ${{{\mathscr {A}}}_{\mathrm{n}}}{\mathrm{= }}\left\{ {1,1,0} \right\}$ if device n offloads some tasks to a D2D helper, and we have ${{{\mathscr {A}}}_{\mathrm{n}}}{\mathrm{= }}\left\{ {1,0,1} \right\}$ if device n chooses the edge server for remote execution. We can get $1 \le {\mathrm{x}}_n + {y_n} + {z_n} \le 2$, with ${{\mathrm{x}}_n},{y_n} \in \{ 0,1\}$.

**Figure 2:**
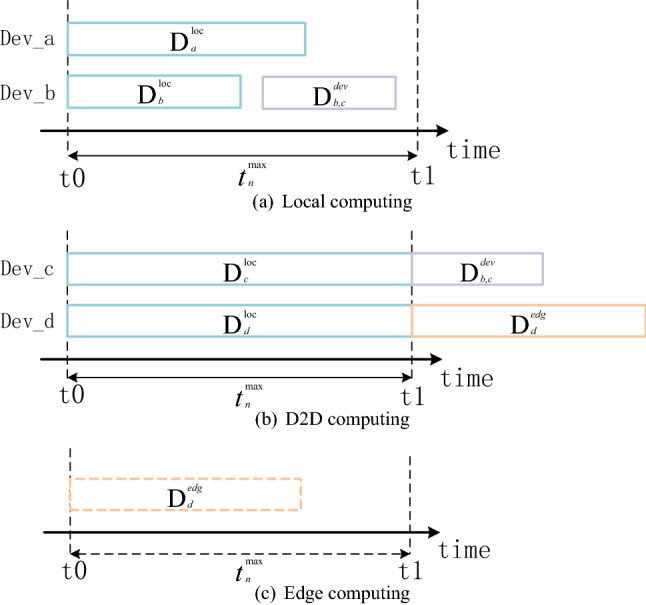
Delays for offloading and computing.

As depicted in Fig. 2, Dev_a, Dev_b, Dev_c and Dev_d correspond to Dev_a, Dev_b, Dev_c and Dev_d in Fig. 1 respectively. From the perspective of the utilization of computing resources, Dev_b in Fig. 2a provides additional computing resources for Dev_c in Fig. 2b, which increases the utilization of resources in Dev_b and enables the task of Dev_c to be completed within the required time. Besides, we use $$ D_{b,c}^{dev}$$ to represent the subtask that Dev_b would execute for Dev_c. The edge server in Fig. 2c provides additional computing resources for Dev_d in Fig. 2b. The edge server can handle a large number of tasks, which are also a factor affecting the processing of the edge server.

We next discuss the overhead of Local computing, Local+D2D computing and Local+D2D+Edge computing in terms of three aspects (delay analysis, energy consumption and computing efficiency).

#### Local computing

Local computing means that a device completes the task locally with its inherent computing capability within a specified time. The time taken by a local computing device is expressed as

2 $$\begin{aligned} T_n^{loc} = {{ C_n^{loc} } \Big / {{f}_n^{loc} }},n \in {{\mathscr {N}}} \end{aligned}$$

where ${f}_n^{loc}$ represents the local computing capability of device n (in CPU cycles/s), and we use ${P}_n^{loc}$ to represent the local processing power of device n (in watt). Energy consumption brought in task processing is mainly studied, so the inherent energy consumption brought by the chip structure is not considered here. The energy consumption can then be expressed as

3 $$\begin{aligned} {E}_n^{loc} = {P}_n^{loc} \left( {{{ C_n^{loc} } \Big / {{f}_n^{loc} }}} \right) ,n \in {{\mathscr {N}}} \end{aligned}$$

We define the computing efficiency(CE) of device n during $t_n^{\max }$ as the proportion of the task handled by the device to the task that could be executed with the inherent computation capacity of the device within this time range. In this way, the local CE can be expressed as

4 $$\begin{aligned} { { {CE}_n^{loc} = C}_n^{loc}} \Big / {{f}_n^{loc} t_n^{\max }},n \in {{\mathscr {N}}} \end{aligned}$$

Our goal is to enhance the CE of local execution devices, namely Dev_a and Dev_b in Cluster1 in Fig. 1. When a device executes some tasks as a helper during $t_n^{\max }$, the CE of the device increases.

#### Local + D2D computing

A device that can’t entirely execute a task within its computation capability needs to request computing resources from nearby devices. When the device finds an adjacent device that can assist in processing partial tasks, the execution mode is changed to Local+D2D mode, and the auxiliary device is a helper. Device n1 represents the helper, and device n2 represents the neighboring device that needs help from device n1, and device n2 offloads the task of size $$ D_{n1,n2}^{dev}$$ to device n1. The delay caused by the D2D communication consists of two parts, the transmission delay of the subtask that needs to be executed by the helper device and the processing delay in the helper. The delay for device n2 and its helper n1 and the energy consumption for device n2 can be expressed as

5 $$\begin{aligned} T_{n2}^{dev}&= {{ D_{n1,n2}^{dev} } \Big / { r_{n1,n2}^{dev} }} + {{ C_{n1,n2}^{dev} } \Big / {{f}_{n1}^{dev} }},n1 \in {{{\mathscr {N}}}_1},n2 \in {{{\mathscr {N}}}_2} \end{aligned}$$

6 $$\begin{aligned} T_{n1,n2}^{helper}&= T_{n1}^{loc} + {{ C_{n1,n2}^{dev} } \Big / {{f}_{n1}^{dev} }},n1 \in {{{\mathscr {N}}}_1},n2 \in {{{\mathscr {N}}}_2} \end{aligned}$$

7 $$\begin{aligned} {E}_{n2}^{dev}&= {P}_{n1,n2}^{com} \left( {{{ D_{n1,n2}^{dev} } \Big / { r_{n1,n2}^{dev} }}} \right) + {P}_{n2}^{idl} \left( {{{ C_{n1,n2}^{dev} } \Big / {{f}_{n1}^{dev} }}{\mathrm{+ }}\left| {{{ D_{n1,n2}^{dev} } \Big / { r_{n1,n2}^{dev} }} - T_{n1}^{loc} } \right| } \right) , n1 \in {{{\mathscr {N}}}_1},n2 \in {{{\mathscr {N}}}_2} \end{aligned}$$

where ${f}_{n1}^{dev}$ represents the computation capability of the helper, ${P}_{n1,n2}^{com}$ represents the transmission power of D2D communication link, ${P}_{n2}^{idl}$ represents idle power of device n2 (in watt). Besides, we define $T_{n1}^{dev'} = {{ C_{n1,n2}^{dev} } \Big / {{f}_{n1}^{dev} }}$ represents the processing delay in the helper device. After the device executes a partial task as a helper, its energy consumption increases and is expressed as

8 $$\begin{aligned} {E}_{n1}^{helper} = {E}_{n1}^{loc} + {P}_{n1}^{loc} \left( {{{ C_{n1,n2}^{dev} } \Big / {{f}_{n1}^{loc} }}} \right) ,n1 \in {{{\mathscr {N}}}_1},n2 \in {{{\mathscr {N}}}_2} .\end{aligned}$$

After executing the additional task as a helper, the computing efficiency of this helper device improves and can be expressed as

9 $$\begin{aligned} {CE}_{n1}^{total}&= {CE}_{n1}^{loc} + {{ D_{n1,n2}^{dev} {\mathrm{Ap}}{{\mathrm{p}}_{n2}}} \Big / {{f}_{n1}^{dev} t_{n1}^{\max } }} \nonumber \\&= {{{ C_{n1}^{loc} } \Big / {{f}_{n1}^{loc} t_{n1}^{\max } }} + C_{n1,n2}^{dev} } \Big / {{f}_{n1}^{dev} t_{n1}^{\max } },n1 \in {{{\mathscr {N}}}_1},n2 \in {{{\mathscr {N}}}_2} .\nonumber \\ \end{aligned}$$

#### Local + D2D + edge computing

Devices that did not choose to offload the subtasks to nearby helper devices would offload their subtasks to the edge server for task execution. The execution mode is modified into Local+D2D+Edge mode. Similar to Local+D2D mode, the delay is also composed of the transmission part and processing part. The delay and energy consumption can be expressed as

10 $$\begin{aligned} T_n^{edg}&= {{ D_n^{edg} } \Big / { r_{n,e}^{edg} }} + {{ C_n^{edg} } \Big / {{f}_n^{edg} }},n \in {{{\mathscr {N}}}_3} \end{aligned}$$

11 $$\begin{aligned} {E}_n^{edg}&= {P}_{n,e}^{com} \left( {{{ D_n^{edg} } \Big / { r_{n,e}^{edg} }}} \right) + {P}_n^{idl} \left( {{{ C_n^{edg} } \Big / {{f}_n^{edg} }}} \right) ,n \in {{{\mathscr {N}}}_3} \end{aligned}$$

where ${P}_{n,e}^{com}$ represents the transmission power of the cellular link. In this paper, the consumption (time and energy) is considered from the perspective of devices using battery power, so it is necessary to consider consumption for the devices. Generally, the edge server with cable power always has enough power to complete the tasks, so the calculation energy consumption of the MEC server is omitted here, similar to the work in^47^. Just like the studies in^48,49^, the transfer of calculated results of time and energy consumption from edge server is neglected in this work, as the calculation results are generally much smaller than the calculated input data. It’s also fit for D2D computing.

Finally, we can get the total time delay and total energy consumption of device n in the current time frame expressed as

12 $$\begin{aligned} T_n&= \max \left\{ { T_n^{loc} x_n , {\max {\mathrm{\{ }} T_{{\mathrm{n1,n}}}^{helper} ,T}_n^{dev} {{\mathrm{\} }}y}_n , T_n^{edg} {z}_n } \right\} n \in {{\mathscr {N}}} n1 \in {{{\mathscr {N}}}_1} \end{aligned}$$

13 $$\begin{aligned} {E}_n&= {E}_n^{helper} x_n + {E}_n^{dev} y_n + {E}_n^{edg} {z}_n,n \in {{\mathscr {N}}} \end{aligned}$$

The total delay for each device can be represented by Eq. (12). $$ T_n$$ is the largest one of the time consumed by local execution, D2D execution and the edge server execution.

### Problem formulation

In this section, we propose an integrated framework of computing offloading and computation resource allocation in D2D–MEC wireless cellular networks. We set ${\Psi _n} = [ {\mathrm{T}}_{\mathrm{n}}^{loc} , {\mathrm{T}}_{\mathrm{n}}^{dev'} , {\lambda {\mathrm{T}}}_{\mathrm{n}}^{edg}]$, ${\mu _n} = [ D_n^{loc} , D_n^{dev} , D_n^{edg}]$, and give the optimization formulation of the problem as follows

$$\begin{aligned} \begin{aligned} ({\mathscr {P}}1): \max&\sum \limits _{\{ {{{\mathscr {A}}}_{\mathrm{n}}},\mu _n ,{f}_n^{edg} \} } {\Psi _n}{{\mathscr {A}}}_n^T/ t_{\mathrm{n}}^{\max }\\ \text{ s.t. }\ (C1):&x_n , y_n ,{z}_n \in \left\{ {0,1} \right\} , n \in {{\mathscr {N}}}\\ (C2):&1 \le {\mathrm{x}}_n + {y_n} + {z_n} \le 2, n \in {{\mathscr {N}}}\\ (C3):&T_{\mathrm{n}} < T_n^{\max } , n \in {{\mathscr {N}}}\\ (C4):&{\mu _n}{{\mathscr {A}}}_n^T = {D_{\mathrm{n}}} , n \in {{\mathscr {N}}} \\ (C5):&\sum \limits _{n \in {{\mathscr {N}}}_3} {{f}_n^{edg} } \le F^{edg} \\ (C6):&{f}_n^{edg} \ge 0, n \in {{\mathscr {N}}}_3 \end{aligned} \end{aligned}$$

${\Psi _n}{{\mathscr {A}}}_n^T/ t_{\mathrm{n}}^{\max }$ represents the proportion of execution time of the device in the maximum time delay of the different modes, and we expect to get a larger one.

Considering the changing trend of ${\mathrm{T}}_{\mathrm{n}}^{loc} / t_{\mathrm{n}}^{\max }$, ${\mathrm{T}}_{\mathrm{n}}^{dev} / t_{\mathrm{n}}^{\max }$ and ${\mathrm{T}}_{\mathrm{n}}^{edg} / t_{\mathrm{n}}^{\max }$ are opposite, we prefer the proportion of the former two to be as high as possible, and the latter is vice versa. Thus, we introduce the negative value $\lambda$ to represent the weight of ${\mathrm{T}}_{\mathrm{n}}^{edg}$. Constraints C1 and C2 guarantee the offloading decisions which meet some conditions. Constraint C3 bounds the maximum delay time of each device. Constraint C4 ensures that all tasks can completely be executed. Constraint C5 is the total computation resource limitation of the edge server. Constraint C6 ensures that each device offloads its task to the edge server and can be allocated to some computation resources. We can observe that ${\mathscr {P}}1$ is a mixed-integer non-linear problem consists of both combinational variables $\{ {{{\mathscr {A}}}_{\mathrm{n}}}\}$ and continuous variables $\{ \mu _n, {f}_n^{edg} \}$ which is hard to resolve. In the next section, we will decompose it into two phases and solve them by heuristic algorithms.

### Problem decomposition and solution

The main challenge in solving ${\mathscr {P}}1$ is that both combinational variables $\{ {{{\mathscr {A}}}_{\mathrm{n}}}\}$ and continuous variables $\{ \mu _n, {f}_n^{edg} \}$ are involved. However, by analyzing the problem, we can successfully divide it into two phases and then solve them individually.

By analyzing these six constraints, we can find that constraints C1 and C2 are about task offloading, constraints C5 and C6 are about the resource allocation of the edge server and constraints C3 and C4 are aimed at the whole task process. The offloading decisions ${{\mathrm{x}}_n},{y_n}{\mathrm{}},{z_n}$ satisfy $1 \le {{\mathrm{x}}_n} + {y_n} + {z_n} \le 2$. We know that task executed via D2D connection is also executed on another helper device and $1 \le {{\mathrm{x}}_n} + {y_n} \le 2$ holds. It has nothing to do with the edge server, which means constraints C2 and C4 can be divided. Based on this, we decompose the task into two parts, executing on the device through D2D communication and executing on the edge server.

#### Task assignment and offloading decision

Since our goal is to maximize the weighted sum of the computing efficiency and the proportion of edge server execution time to the total time of all devices, the main limitations of ${\mathscr {P}}1$ include the task offloading strategy and the limited computation resources of the edge server. In the first phase, we decompose the task offloading strategy and device computing efficiency acquired from ${\mathscr {P}}1$ and its mathematical formula can be expressed as

$$\begin{aligned} \begin{aligned} ({\mathscr {P}}2): \max&\sum \limits _{\{ {{{\mathscr {A}}}_{\mathrm{n}}},\mu _n \} } { {CE}_n^{total} }\\ \text{ s.t. }\ (C1) \sim&(C2)\\ (C3)^1:&\max \left\{ { T_n^{loc} x_n , {\max {\mathrm{\{ }} T_{{\mathrm{n1,n}}}^{helper} ,T}_n^{dev} {{\mathrm{\} }}y}_n } \right\} \le T_n^{\max } , n \in {{\mathscr {N}}}, n1 \in {{{\mathscr {N}}}_1}\\ (C4)^1:&D_n^{loc} + D_n^{dev} {\mathrm{= }} D_{\mathrm{n}} , n \in {{\mathscr {N}}}_2 \\ \end{aligned} \end{aligned}$$

According to Eq. (9), the computing efficiency obtained from the task execution is ${{{ C_n^{loc} } \Big / {{f}_n^{loc} t_n^{\max } }} + C_{n,n2}^{dev} } \Big / {{f}_n^{dev} t_n^{\max } }$, according to Eq. (2), the local execution time is $T_n^{loc} = {{ C_n^{loc} } \Big / {{f}_n^{loc} }}$, and we know $T_n^{dev'} = {{ C_{n,n2}^{dev} } \Big / {{f}_n^{dev} }}$. It is easy to find that $CE_n^{total} = {\mathrm{T}}_{\mathrm{n}}^{loc} / t_{\mathrm{n}}^{\max } + {\mathrm{T}}_{\mathrm{n}}^{dev'} / t_{\mathrm{n}}^{\max }$, they are formulated from two aspects, i.e., computation resources occupation and time occupation.

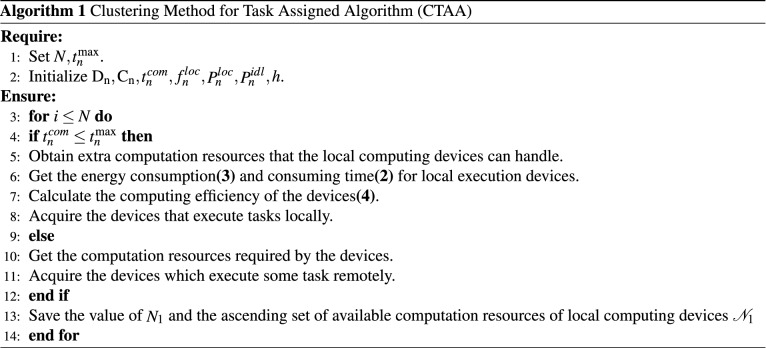



We transform the constraint (C3)$\sim$(C4) into $(C3)^1\sim (C4)^1$, because the device that needs help belongs to ${{\mathscr {N}}}_2$. Only the device that requires additional resources via D2D connection is considered, and its task is executed locally or executed through a helper. The total delay of these devices is the largest one of the local execution delay, the delay of the device that executes D2D computing and the helper execution delay, namely constraint $(C3)^1$.

According to whether the devices can complete their tasks within the required time, we divide the devices into two clusters: local computing and remote computing. The proposed clustering method for the task-assigned algorithm (CTAA) is described in Algorithm 1. Local computing devices may have excess computing resources to provide for another device, while remote computing devices have reached the maximum computing efficiency when performing local computing. Then, the low complexity dichotomy algorithm (LCDA) described in Algorithm 2 is used to get the set of devices that execute the remaining tasks in the nearby helper devices and the edge server respectively.

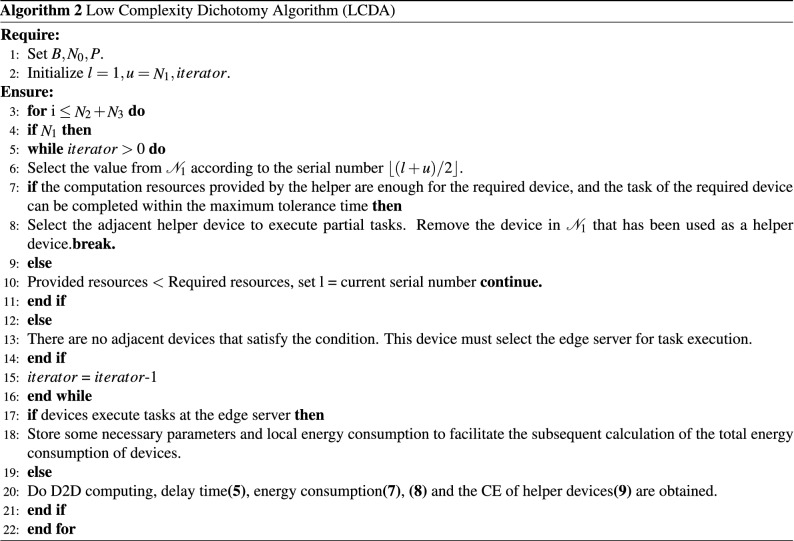



In Algorithm 2, the choice of the adjacent helper is reflected in using LCDA to match the computation resources required by the device and provided by the helper device to achieve the best matching. The device with enormous task requirements can match the corresponding helper device and vice versa. It’s the sub-optimal but best solution because the optimal solution requires a perfect match, which is bound to incur a lot of extra costs.

#### Computation resource allocation

The second phase considers the allocation of computing resources at the edge server to minimize computing time. We can express the problem as

$$\begin{aligned} \begin{aligned} ({\mathscr {P}}3): \min&\sum \limits _{\{ {f}_n^{edg} \} } {\lambda {\mathrm{T}}_{\mathrm{n}}^{edg} } \!\Big / \!{ t_n^{\max } }\\ \text{ s.t. }\ (C3)^2:&T_n^{edg} \le T_n^{\max } , n \in {{{\mathscr {N}}}_3}\\ (C4)^2:&D_n^{loc} + D_n^{edg} {\mathrm{= }} D_{\mathrm{n}} ,n \in {{\mathscr {N}}}_3 \\ (C5) \sim&(C6)\\ \end{aligned} \end{aligned}$$

We convert (C3)$\sim$(C4) to $(C3)^2$ $\sim$ $(C4)^2$. The devices offloaded to the edge server are in ${{\mathscr {N}}}_3$, which does not involve helper execution, so this conversion is reasonable. In this round of task execution, the maximum delay of all devices offloaded to the edge server is the same, i.e., $T_{\mathrm{n}}^{\max }$. We need to ensure the execution time of each device is not over the maximum time delay, namely constraint $(C3)^2$. We set the computing resources allocated to the devices that execute tasks at the edge server as ${\{ f}_1^{edg} ,{f}_1^{edg} ,......,{f}_{ N_3 }^{edg} \}$. The total amount of computing resources on the edge server is certain. Namely, the sum of $\sum \limits _{n \in {\mathcal{N}_3}} {{f}_n^{edg} }$ is a certain value, ${\mathrm{F}}^{{\mathrm{fog}}}$. To find the optimal solution, we only need to find the minimum value of $\sum \nolimits _{n \in {\mathcal{N}_3}} { T_n^{edg} }$, i.e., $\sum \nolimits _{n \in {{{\mathscr {N}}}_3}} { T_n^{edg} } = {{ {\mathrm{C}}_1^{{\mathrm{edg}}} } \Big / {{f}_1^{{\mathrm{edg}}} }} + {{ {\mathrm{C}}_2^{{\mathrm{edg}}} } \Big / {{f}_2^{{\mathrm{edg}}} }} + ...... + {{ {\mathrm{C}}_{ N_3 }^{{\mathrm{edg}}} } \Big / {{f}_{ N_3 }^{{\mathrm{edg}}} }}$. To ensure that the edge server resources are utilized effectively and all tasks are executed in the shortest time, we transform problem ${{\mathscr {P}}}_3$ to obtain the resources allocation ratio which meets the minimum requirements. Let this ratio be $F_1^{{\mathrm{edg}}} , F_2^{{\mathrm{edg}}} ,......, F_{ N_3 }^{{\mathrm{edg}}}$, and we set ${f}_1^{edg} = \eta F_1^{{\mathrm{edg}}} ,{f}_2^{edg} = \eta F_2^{{\mathrm{edg}}} ,......,{f}_{ N_3 }^{edg} = \eta F_{ N_3 }^{{\mathrm{edg}}}$, where $\eta = {{ F^{fog} } \Bigg / {\sum \nolimits _{n \in {{{\mathscr {N}}}_3}} { F_n^{edg} } }}$. When each device is assigned specific resources, we can transform $\sum \nolimits _{n \in {{{\mathscr {N}}}_3}} { T_n^{edg} }$ into

$$\begin{aligned} {{ {\mathrm{C}}_1^{{\mathrm{edg}}} } \Big / { F_1^{{\mathrm{edg}}} }} + {{ {\mathrm{C}}_2^{{\mathrm{edg}}} } \Big / { F_2^{{\mathrm{edg}}} }} + ...... + {{ {\mathrm{C}}_{ N_3 }^{{\mathrm{edg}}} } \Big / { F_{ N_3 }^{{\mathrm{edg}}} }} \end{aligned}$$

Then we add and subtract the sum of coefficients $\sum \nolimits _{n = 1}^{ N_3 } { F_n^{{\mathrm{edg}}} }$ respectively, and the value of the formula doesn’t change. Utilizing means inequality to get the solution

$$\begin{aligned}&{{ {\mathrm{C}}_1^{{\mathrm{edg}}} } \Big / { F_1^{{\mathrm{edg}}} }} + {{ {\mathrm{C}}_2^{{\mathrm{edg}}} } \Big / { F_2^{{\mathrm{edg}}} }} + \cdots  \cdots+ {{ {\mathrm{C}}_{ N_3 }^{{\mathrm{edg}}} } \Big / { F_{ N_3 }^{{\mathrm{edg}}} }}=  {{ {\mathrm{C}}_1^{{\mathrm{edg}}} } \Big / { F_1^{{\mathrm{edg}}} }} \\&\qquad + F_1^{{\mathrm{edg}}} + {{ {\mathrm{C}}_2^{{\mathrm{edg}}} } \Big / { F_2^{{\mathrm{edg}}} }} + F_2^{{\mathrm{edg}}} +  \cdots \cdots + {{ {\mathrm{C}}_{ N_3 }^{{\mathrm{edg}}} } \Big / { F_{ N_3 }^{{\mathrm{edg}}} }}+  F_{ N_3 }^{{\mathrm{edg}}} - \sum \limits _{n = 1}^{ N_3 } { F_n^{{\mathrm{edg}}} }\\&\quad \ge 2\sqrt{ {\mathrm{C}}_1^{{\mathrm{edg}}} } + 2\sqrt{ {\mathrm{C}}_2^{{\mathrm{edg}}} } + \cdots \cdots + 2\sqrt{ {\mathrm{C}}_{ N_3 }^{{\mathrm{edg}}} } - \sum \limits _{n = 1}^{ N_3 } { F_n^{{\mathrm{edg}}} } \end{aligned}$$

According to the property of inequality, we get

$$\begin{aligned} F_1^{{\mathrm{edg}}} = \sqrt{ {\mathrm{C}}_1^{{\mathrm{edg}}} } , F_2^{{\mathrm{edg}}} = \sqrt{ {\mathrm{C}}_2^{{\mathrm{edg}}} } ,\ldots\ldots, F_n^{{\mathrm{edg}}} = \sqrt{ {\mathrm{C}}_{ N_3 }^{{\mathrm{edg}}} } \end{aligned}$$

Finally, we can get the computing resources allocated to each device executing the task on the edge server. The specific algorithm is described in Algorithm 3.

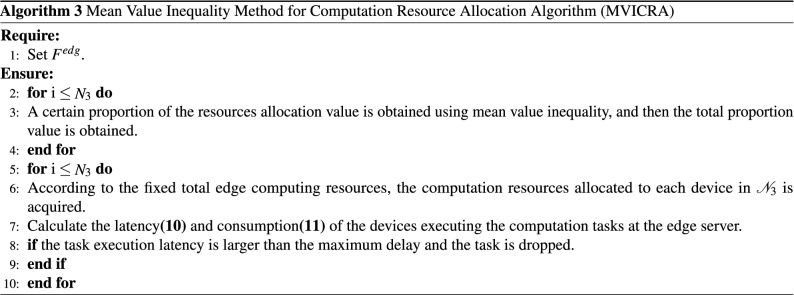



## Simulation results

In this section, simulation results of the proposed D2D–MEC system are presented to verify the performance enhancement. The channel power gain in the D2D–MEC system is modeled as ${\mathrm{h}} = {10}^{ - 3} { d^{ - \zeta }}\phi$, where $d \sim u(0.2,30)$(in m) represents the distance between the two communication terminals, $\zeta$ represents the path-loss exponent and is assumed to be 2.5^23^, and $\phi$ is small-scale fading and $\phi \sim CN(0,1)$ is an independent and identically distributed circularly symmetric complex Gaussian vector with zero mean and covariance one^27^. The major simulation parameters employed in the simulations, unless otherwise stated, are summarized in Table 3.

### Task execution result

**Figure 3:**
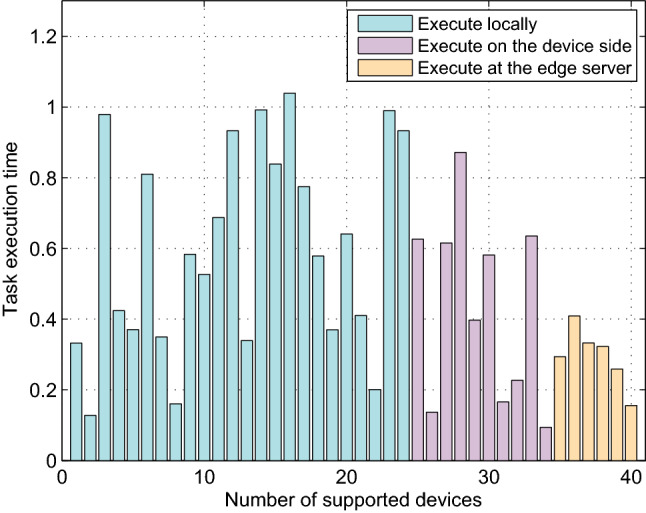
The task execution time versus number of supported devices in the system (N = 40).

**Figure 4:**
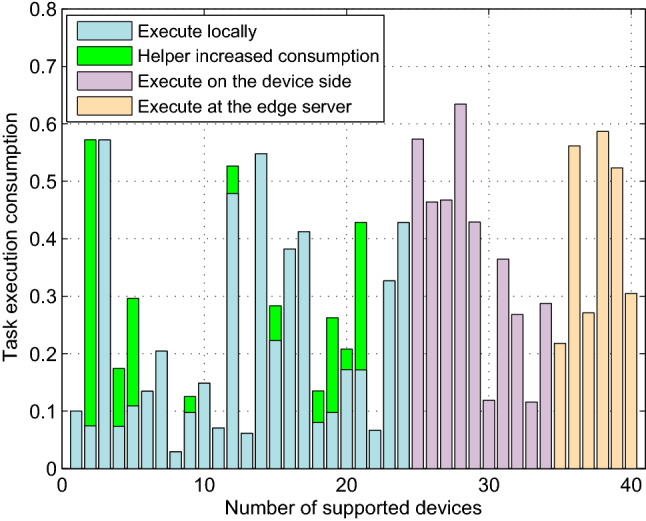
The task execution consumption versus number of supported devices in the system (N = 40).

The simulation is carried out to verify the rationality of the proposed algorithm from two aspects, i.e., task execution time and task execution energy consumption of each device, as shown in Figs. 3 and 4. In Fig. 3, the number and the access ratio of devices executed locally, offloaded to a helper via D2D connection and offloaded to the edge server via cellular connection are (24, 10, 6), (0.6, 0.25, 0.15) respectively. The number of devices executed locally is significantly higher than that executed remotely and the tasks’ execution time at the edge server is relatively less because they are not heavy. In addition, all tasks are completed within the maximum tolerance time.

**Table 3:** Simulation parameters.

Parameter	Value
Total number of devices, N	40
Delay tolerance, T	1.1s
Data size of task, ${\mathrm{D}}_{\mathrm{n}}$	[0.1, 2] Mbits
Sub-channel bandwidth, B	0.5 MHz^39^
Computation resources of the device, ${{f}_n^{loc} }$	[0.5, 2] $\times {10}^9$ CPU cycles/s^39^
Power density of the noise, $$ {\mathrm{N}}_0$$	$$ {10}^{ - 8}$$^23^
Transmit power, ${P}_{\mathrm{n}}^{com}$	0.15 W^23^
Power of device n in local/idle processing, ${P}_n^{loc }, {P}_n^{idl }$	[0.1, 0.5] W, [0.001, 0.01] W^50^
Required CPU cycles per bit, ${\mathrm{App}}_{\mathrm{n}}$	[500,2000]CPU cycles/bit
Edge computation resource, ${\mathrm{F}}^{{\mathrm{fog}}}$	$40 \times {10}^9$ CPU cycles/s^39^

Task execution delay and consumption are two important factors affecting the performance of a model. As shown in Fig. 4, the energy consumption of each device and the incremental energy consumption of helper devices are given. The average energy consumption of local computing devices is lower than that of remote computing devices. Devices have fluctuated time and consumption to execute tasks locally, which depends on their performance and the size of the assigned tasks. The results show that the proposed resource allocation model and task offloading algorithm can ensure that each device could complete the assigned task with less energy consumption within the specified time delay.

### Task mode comparison

To increase the computing efficiency and the access rate of the devices, improve the completion rate of the tasks in the system, three task execution modes are adopted, namely local computing, D2D computing and edge server computing. We measure the effectiveness of the proposed algorithm in terms of the number of devices existing on the system, the task size assigned to a single device and the task execution delay, then carry out the comparison of the three modes. The results are averaged over 1000 independent experiments to ensure the scientific nature of the simulation.

**Figure 5:**
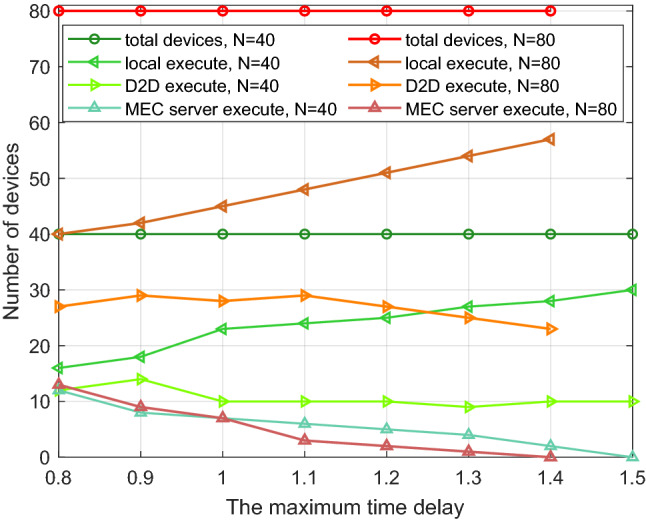
The number of devices versus the maximum time delay of different kinds.

We set the number of devices to 40 and 80 respectively. When the number of devices is the same and the maximum delay ranges from 0.8 to 1.5 s, we conduct a statistical simulation of device access in the three modes. As Fig. 5 shows, the trend is the same. We analyze the situation when the total number of devices is 40. When the delay is the minimum one (i.e., 0.8 s), the number of devices that execute tasks locally reaches the minimum value. The number of devices that offload tasks to helpers via D2D connection is relatively smooth cause only the devices with high performance allocated relatively small tasks are likely to execute additional tasks as helpers and these devices are relatively constant. In addition, the number of devices that execute tasks remotely is the maximum at this time, so is that of offload tasks to the edge server. With the increase of time delay, the number of devices that can complete tasks locally increase slowly, while that of offload tasks remotely decrease. The utilization of computing resource at the edge server is reduced, so is the number of devices executed on it. The remaining computing resources on the edge server can be used in other parts of the cellular network.

**Figure 6:**
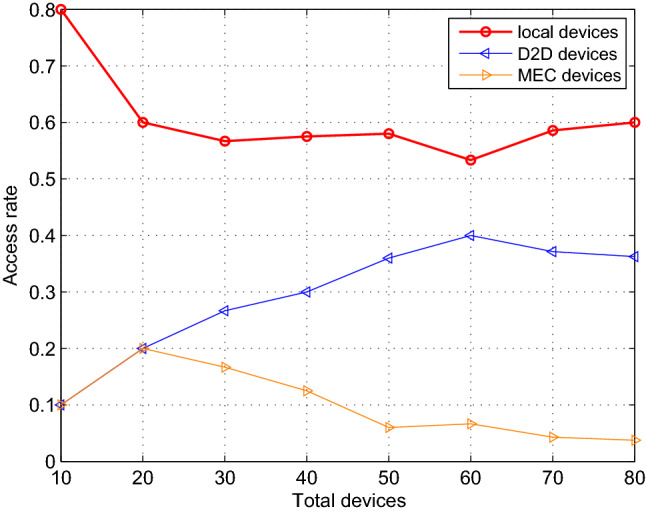
The access rate versus the number of total devices in the system.

**Figure 7:**
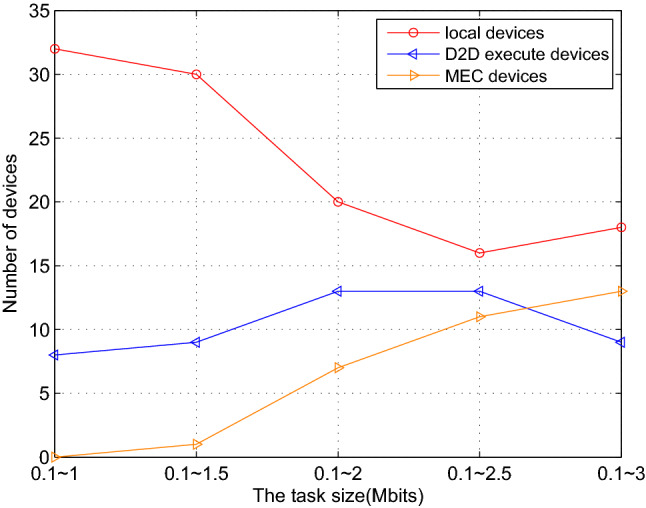
The number of devices versus the task size of different kinds.

The introduction of D2D communication improves the computing efficiency of the device and relieves the computing pressure of the edge server effectively. In addition, if the latency is too small, most of the devices can not complete their tasks locally, which causes the congestion of the connected links and the resources allocated by the edge server may not be sufficient to complete the task.

As shown in Fig. 6, with the number of devices ranging from 10 to 80 in the system under the three modes, we conduct a statistical analysis on the access rate of devices. The performance configuration of the devices is shown in Table 3. When the total number of devices is less than 20, the number of local computing devices is larger. As the devices’ number increases and ranges from 20 to 50, D2D mode and the edge mode change in opposite directions. At this time, the number of devices accessing D2D mode increases steadily. When the number of devices is 40, the corresponding access rate of the three modes is (0.58, 0.3, 0.13) respectively, which has the same conclusion obtained in Fig. 3. When the total number of devices is greater than 50, the device access rate tends to be stable in the three modes. Besides, some insightful results can be obtained via simulation parameters from Table 3. The ranges of ${\mathrm{D}}_{\mathrm{n}}$ and ${\mathrm{Ap}}{{\mathrm{p}}_{\mathrm{n}}}$ are [0.1, 2] Mbits and [500, 2000] CPU Cycles/bit respectively, the tolerance time is 1.1s and the computation resource of a device fn is [0.5, 2] CPU cycles/s. For convenience of description, the lower and upper limits of Dn and Appn are denoted by ${\mathrm{D}}_{\mathrm{n}}^{\mathrm{l}}$ and ${\mathrm{D}}_{\mathrm{n}}^{\mathrm{h}}$, ${\mathrm{App}}_{\mathrm{n}}^{\mathrm{l}}$ and ${\mathrm{App}}_{\mathrm{n}}^{\mathrm{h}}$ respectively. The joint distribution of ${\mathrm{D}}_{\mathrm{n}}$ and ${\mathrm{Ap}}{{\mathrm{p}}_{\mathrm{n}}}$, namely the task assigned to each device is uniformly distributed on the rectangle ${\mathrm{S}} = \left\{ {\left( {{\mathrm{x,y}}} \right) |{\mathrm{D}}_{\mathrm{n}}^{\mathrm{l}} \le {\mathrm{x}} \le {\mathrm{D}}_{\mathrm{n}}^{\mathrm{h}},{\mathrm{App}}_{\mathrm{n}}^{\mathrm{l}} \le {\mathrm{y}} \le {\mathrm{App}}_{\mathrm{n}}^{\mathrm{h}}} \right\}$. When $\left( {{\mathrm{x,y}}} \right) \in {\mathrm{S}}$ , the joint probability density is ${\mathrm{f}}\left( {{\mathrm{x,y}}} \right) = {1 \Big / {\left( {\left( {{\mathrm{D}}_{\mathrm{n}}^{\mathrm{h}} - {\mathrm{D}}_{\mathrm{n}}^{\mathrm{l}}} \right) \left( {{\mathrm{App}}_{\mathrm{n}}^{\mathrm{h}} - {\mathrm{App}}_{\mathrm{n}}^{\mathrm{l}}} \right) } \right) }}$ , when $\left( {{\mathrm{x,y}}} \right) \notin {\mathrm{S}}$ , ${\mathrm{f}}\left( {{\mathrm{x,y}}} \right) = 0$ . Since the range of task which can be executed locally by the device follows the uniform distribution of $[{\mathrm{Tsk}}_{\mathrm{n}}^{\mathrm{l}},{\mathrm{Tsk}}_{\mathrm{n}}^{\mathrm{h}}]$, where ${\mathrm{Tsk}}_{\mathrm{n}}^{\mathrm{l}}=0.5*1.1*{10^9}$ and ${\mathrm{Tsk}}_{\mathrm{n}}^{\mathrm{h}}=2*1.1*{10^9}$. By solving the probability

$$\begin{aligned}&{\mathrm{P}}\left( {{\mathrm{S}}\left( {{\mathrm{X,Y}}} \right) \le {\mathrm{Tsk}}_{\mathrm{n}}^{\mathrm{h}}} \right) - {\mathrm{P}}\left( {{\mathrm{S}}\left( {{\mathrm{X,Y}}} \right) \le {\mathrm{Tsk}}_{\mathrm{n}}^{\mathrm{l}}} \right) {\mathrm{= }}\left( {1 - \int _{{{{\mathrm{Tsk}}_{\mathrm{n}}^{\mathrm{h}}} \Big / {{\mathrm{App}}_{\mathrm{n}}^{\mathrm{h}}}}}^{{\mathrm{D}}_{\mathrm{n}}^{\mathrm{h}}} {\int _{{{{\mathrm{Tsk}}_{\mathrm{n}}^{\mathrm{h}}} \Big / {\mathrm{x}}}}^{{\mathrm{App}}_{\mathrm{n}}^{\mathrm{h}}} {{\mathrm{f}}\left( {{\mathrm{x,y}}} \right) } } } \right) \\&\quad - \left( {1 - \int _{{{{\mathrm{Tsk}}_{\mathrm{n}}^{\mathrm{l}}} \Big / {{\mathrm{App}}_{\mathrm{n}}^{\mathrm{h}}}}}^{{\mathrm{D}}_{\mathrm{n}}^{\mathrm{h}}} {\int _{{{{\mathrm{Tsk}}_{\mathrm{n}}^{\mathrm{l}}} \Big / {\mathrm{x}}}}^{{\mathrm{App}}_{\mathrm{n}}^{\mathrm{h}}} {{\mathrm{f}}\left( {{\mathrm{x,y}}} \right) } } } \right) \\&\quad = \left( {\mathrm{Tsk}}_{\mathrm{n}}^{\mathrm{h}} - {\mathrm{Tsk}}_{\mathrm{n}}^{\mathrm{l}}{\mathrm{+ }}\left( {{\mathrm{Tsk}}_{\mathrm{n}}^{\mathrm{h}} - {\mathrm{Tsk}}_{\mathrm{n}}^{\mathrm{l}}} \right) {\mathrm{*}}\ln \left( {{\mathrm{D}}_{\mathrm{n}}^{\mathrm{h}}*{\mathrm{App}}_{\mathrm{n}}^{\mathrm{h}}} \right) + {\mathrm{Tsk}}_{\mathrm{n}}^{\mathrm{l}}*\ln \left( {{\mathrm{Tsk}}_{\mathrm{n}}^{\mathrm{l}}} \right) \right. \\&\left. \qquad - {\mathrm{Tsk}}_{\mathrm{n}}^{\mathrm{h}}*\ln \left( {{\mathrm{Tsk}}_{\mathrm{n}}^{\mathrm{h}}} \right) \right) \Big / \left( {\left( {{\mathrm{D}}_{\mathrm{n}}^{\mathrm{h}} - {\mathrm{D}}_{\mathrm{n}}^{\mathrm{l}}} \right) \left( {{\mathrm{App}}_{\mathrm{n}}^{\mathrm{h}} - {\mathrm{App}}_{\mathrm{n}}^{\mathrm{l}}} \right) } \right) \end{aligned}$$

We can get the ratio of the devices that execute tasks locally to be 0.65 accordingly. When the total number of devices is 40 and 80, the theoretical optimal value of the local execution devices should be 26 and 52 respectively. As shown in Fig. 5, the actual measured value is 24 and 49 respectively, and the matching degree reaches 93.27%, which is within the tolerance and consistent with our conclusion.

We extended the task size range from [0.1, 1] to [0.1, 3] Mbits to conduct statistical analysis on the number of devices in the three modes. Different task size will also affect the task execution process of each device. As shown in Fig. 7, when the task size is small, local mode and D2D mode can finish all tasks in the system. As the maximum task cap increased, some devices need to execute larger tasks, the performance of local devices limits the number of devices that can execute tasks locally. The increasing number of devices that can’t execute tasks locally affects the devices’ number that execute tasks through D2D mode. The number of devices executing tasks in D2D mode increases slightly and remains relatively stable overall. At this time, D2D mode can no longer improve the overall performance of the system. The number of devices executed on the edge server is increasing to relieve system stress. Compared with traditional D2D communication, Local + D2D + MEC mode has more significant advantages in processing tasks.

### Task algorithm optimization

**Figure 8:**
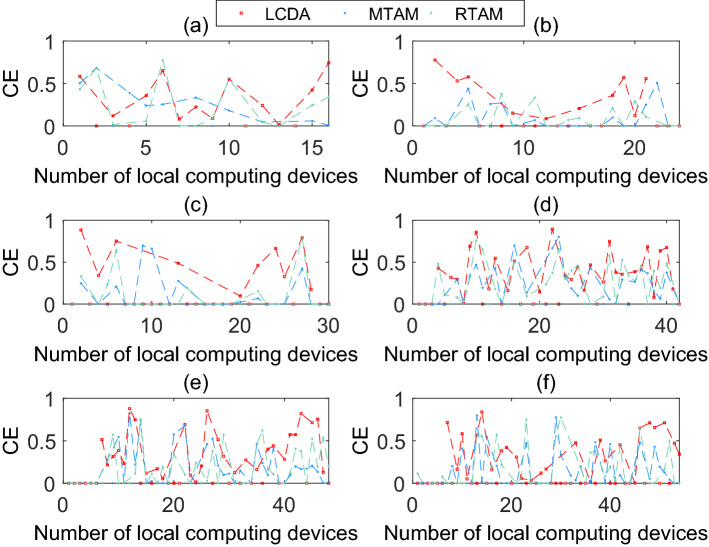
Computing efficiency versus the number of local computing devices of different kinds: (**a**) The number of devices(NUM) and the maximum delay(DEY) is 40 and 0.8s, (**b**) The NUM and DEY is 40 and 1.1s, (**c**) The NUM and DEY is 40 and 1.5 s, (**d**) The number of devices(NUM) and the maximum delay(DEY) is 80 and 0.9 s, (**e**) The NUM and DEY is 80 and 1.1s, (**f**) The NUM and DEY is 80 and 1.3 s.

The mode selection algorithm we proposed in the D2D–MEC system is the low complexity dichotomy algorithm (LCDA), which we compared with two baseline algorithms, namely the maximum task assignment method (MTAM) and the random task assignment method (RTAM). The MTAM algorithm allows a device to select one neighboring device as a helper to provide the largest computation capability, which causes the device that selects previously has a higher hit rate, while that of selects afterward has a lower probability to offload its task. The RTAM algorithm randomly selects one adjacent device as a helper to match the device, i.e., the device that can provide sufficient computing capability. Fig. 8 shows how the proposed algorithm improves the computing efficiency of the local computing devices compared with the other two benchmark algorithms. Fig. 8a–c and d–f show the improvement of computing efficiency of local computing devices when the number of devices is 40 and 80 and the maximum delay is (0.8 s, 1.1 s, 1.5 s) and (0.9 s, 1.1 s, 1.3 s) respectively. Table 4 shows the number of helper devices and the average CE rate under the three algorithms in the six situations mentioned above.

When the devices’ number is fixed, taken 40 as an example, the CE of the device which needs to be offloaded reaches the maximum value, so only the local execution devices are analyzed in the figure. As the delay increases, the total number of local execution devices also increases. The number of devices in Fig. 8a–c is (16, 24, 30). For the convenience of observation, the CE depicted in the figure removes the local CE. It represents the incremental CE of helpers. The number of nodes in the figure represents the number of helpers, and the amplitude represents the increased value of CE. The overall improvement in the proposed algorithm is superior to the other two algorithms.

**Table 4:** The number of helper devices and the average CE rate in different parameters.

Picture number	(a)	(b)	(c)	(d)	(e)	(f)
Total helper devices	16	24	30	42	48	54
Parameter specification	N=40			N=80		
	0.8 s	1.1 s	1.5 s	0.9 s	1.1 s	1.3 s
**The number of helper devices**						
LCDA	12	10	10	29	29	25
MTAM	10	20	17	27	41	43
RTAM	13	21	19	31	41	43
**Average CE rate**						
LCDA	34	39.4	49.8	42.1	39.8	37.1
MTAM	27	9.9	15.2	26.2	18.6	15.2
RTAM	25	8.3	12.4	29	18	17.6

Table 4 shows the number of helper devices and the percentage of improved efficiency under the three algorithms when the total number of devices is 40 and 80 respectively and the maximum time delay is (0.8 s, 1.1 s, 1.5 s) and (0.9 s, 1.1 s, 1.3 s) respectively. The comparison results show that the number of helper devices of the proposed algorithm is relatively small, but the average CE rate is significantly higher than the other two algorithms.

**Figure 9:**
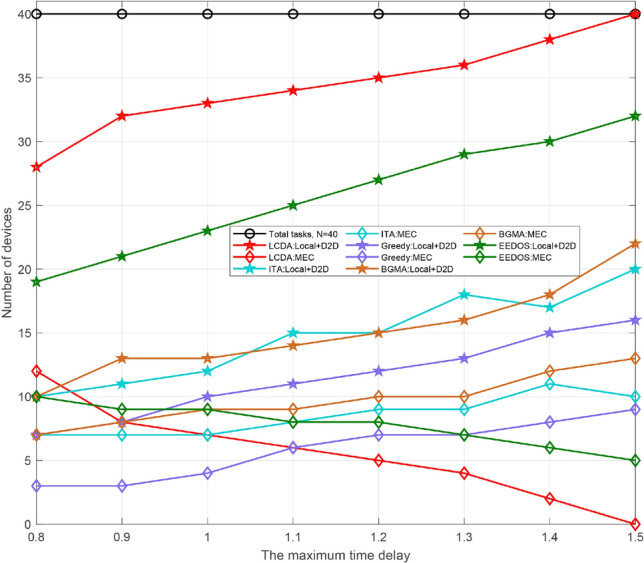
The number of devices versus the maximum time delay of different modes (N = 40).

**Figure 10:**
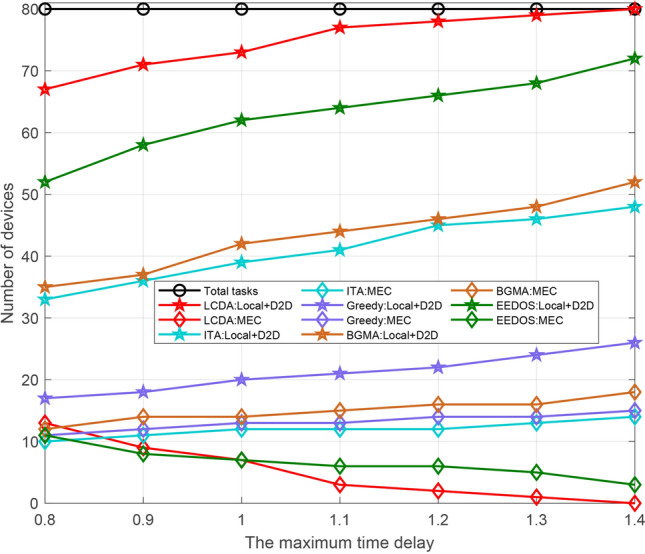
The number of devices versus the maximum time delay of different modes (N = 80).

### Model comparison results

The above simulation results comprehensively analyze the performance of the D2D–MEC system and show that the proposed algorithm can effectively improve the computing efficiency of the devices, enhance the capacity and increase the number of access devices for the system. To further verify the performance of the algorithm, we compare the proposed algorithm with the traditional D2D–MEC algorithm, including the improved greedy algorithm^41^, the initial task assignment algorithm (ITA)^25^, bipartite graph matching algorithm (BGMA)^14^, and energy-efficient and delay-aware offloading scheme (EEDOS)^32^. Besides, the comparison is under our proposed scenarios. The completed tasks of the five algorithms under different execution modes are illustrated in Figs. 9 and 10. Since each device is assigned a task, the devices number of the system is equal to the number of tasks. The devices number in Figs. 9 and 10 is 40 and 80 respectively and the maximum delay is 0.8–1.5 s and 0.8–1.4 s respectively.

As an example, Fig. 9 compares the total number of devices executed through local computing and D2D computing with the number of devices executed on the edge server under different time delays. As shown in the figure, the LCDA algorithm, which gives priority to local computing and D2D computing, can complete all tasks within the specified time delay, while the other four algorithms cannot. When the delay is short, the LCDA algorithm requires more computing resources from the edge server and the EEDOS algorithm has the same trend, while the other algorithms have no significant change in demand for the edge server.

**Figure 11:**
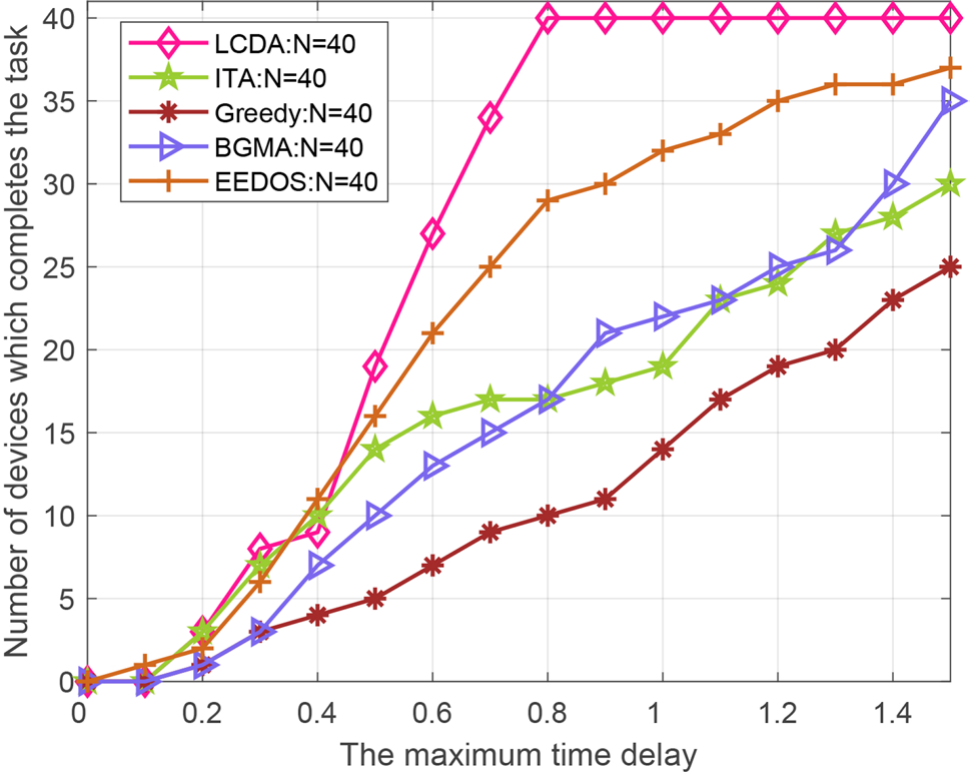
The number of devices that can complete the task versus Maximum delay of different modes (N = 40).

**Figure 12:**
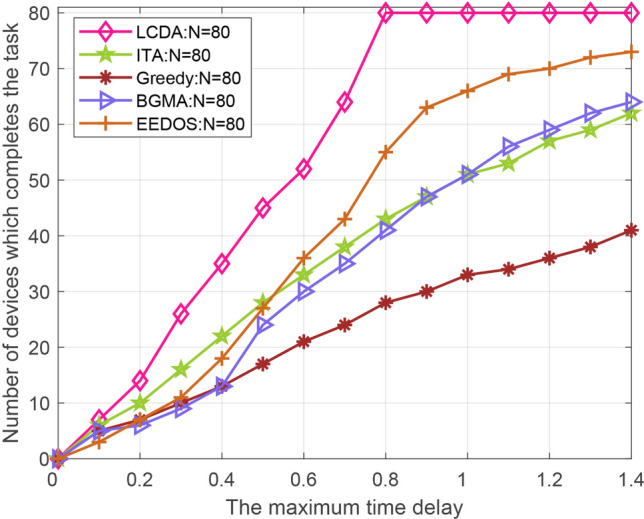
The number of devices that can complete the task versus Maximum delay of different modes (N = 80).

The number of completed tasks of the five algorithms is compared. The number of devices is set to 40 and 80, and the delay constraint is set to 0–1.4 s respectively. Accordingly, we can acquire the total number of tasks completed by the five algorithms in each time delay. Figures 11 and 12 shows that the proposed LCDA algorithm is better than the other four algorithms. With the increase of time delay, the total number of completed tasks of the LCDA algorithm is significantly larger than the other four algorithms. When the delay time reaches 0.8s, all devices under the LCDA algorithm can complete their tasks and remain stable in the following time delay, while at this point, the other four algorithms still can’t complete all tasks.

In conclusion, compared with some traditional D2D–MEC resource allocation algorithms, the LCDA algorithm is superior under the proposed scenarios. The reason for the better performance of the proposed algorithm is that the local and edge server computation resources are considered together and the partial offloading strategy is used in the model. Compared with the proposed algorithm, the improved greedy algorithm only considers binary offloading and idle helper devices, the ITA algorithm ignores the partial offloading strategy, the BGMA algorithm does not take the computing resources of the BS into account, and the EEDOS algorithm only considers the help from idle devices but ignores the high-performance devices with little tasks to be handled.

### Complexity analysis

In this section, we briefly analyze the complexity of the proposed algorithm in two phases, i.e., the computation offloading strategy and D2D computing efficiency acquired in Phase ${{\mathscr {P}}}_2$ and edge server resource allocation in Phase ${{\mathscr {P}}}_3$, where we examine the complexity of solutions to the two phases respectively. In addition, in the proposed scheme, Problem ${{\mathscr {P}}}_2$ can be divided into two sub-problems. The devices are divided into two clusters through Algorithm 1, the time complexity is O(*N*), where *N* represents the number of devices involved in resource allocation. The goal of Algorithm LCDA is to select the helper device and calculate its CE. The time complexity of LCDA is O($\log {N^1}$), where $N^1$ is the number of devices that have been matched in the system, and in the best and the worst case, the complexity is O($N^1$) and O(${iterator}^{{N^1}}$) respectively. According to the fifth line of Algorithm 2, the iterations to find the matching device is set to *iterator*, and $N^1$ is the number of matched devices. When devices can complete their tasks on time but can only provide fewer resources, those that need help need a lot of resources in this period, in this extreme case, the worst computational complexity is attained. The problem of resource allocation of the edge server is solved in ${{\mathscr {P}}}_3$, which can be completed in polynomial time. Accordingly, the overall computational complexity is O(*N*). Compared with the other four traditional D2D–MEC algorithms, the time complexity of the greedy algorithm is O($N^2$), that of ITA algorithm is O($N^{N+2}$) and that of BGMA algorithm is O($TN^3$), which are related to the time delay, and EEDOS algorithm is in a long polynomial. Therefore, the scheme proposed in this paper has a low computational complexity.

## Conclusions

This paper proposes a multi-user D2D–MEC system to improve the computing efficiency of devices, where each device includes a task with a variable length to execute within a specified delay. The devices choose to execute tasks locally unless they are unable to complete them on time and offload some tasks to nearby devices with ample computing resources via D2D execution or an edge server. Firstly, a mixed-integer non-linear problem is presented to maximize the computing efficiency of the system. Then, we resolve it by dividing it into two phases. Specifically, according to the local computation priority, the first phase is to divide a task into local execution and remote execution according to Algorithm 1. The nearby helpers are first selected by the remote executing devices to offload their tasks through Algorithm 2, then an offloading strategy can be obtained by solving the problem. The assignment of computing resources in the edge server is considered in the second phase, and the assignment scheme is obtained through Algorithm 3. Numerical simulation results show that, compared with some traditional D2D–MEC algorithms, the number of access devices and completed tasks can be effectively improved through the proposed algorithm. Further, the task execution efficiency is improved, and a superior performance is achieve with a lower complexity.

Through the combination of D2D communication technology and MEC, computing and spectrum resources are expanded and a large-scale access of devices is increased. This is an application scenario of 5G, providing low-latency service for computation-intensive tasks of mobile terminals, which belongs to the technical field of task offloading in the D2D–MEC system. In addition, ICT technology, as the integration of IT(MEC) and CT(D2D) technology, can be used for infrastructure construction in the smart city of 6G. In future work, ICT, digital twin and blockchain technologies will be applied to the Internet of Vehicles field, which can further promote the research on resource management and task offloading area in multi-link cooperative transmission and secure transmission. Our works have theoretical guiding significance for the subsequent research.
